# Lycium barbarum polysaccharide alleviates dextran sodium sulfate-induced inflammatory bowel disease by regulating M1/M2 macrophage polarization via the STAT1 and STAT6 pathways

**DOI:** 10.3389/fphar.2023.1044576

**Published:** 2023-04-18

**Authors:** Juan Wang, Huiying Gao, Yuan Xie, Peng Wang, Yu Li, Junli Zhao, Chunlin Wang, Xin Ma, Yuwen Wang, Qinwen Mao, Haibin Xia

**Affiliations:** ^1^ Laboratory of Gene Therapy, Department of Biochemistry, College of Life Sciences, Shaanxi Normal University, Xi’an, China; ^2^ Department of Pathology, School of Basic Medical Science, Ningxia Medical University, Yinchuan, Ningxia, China; ^3^ Department of Pathology, Huntsman Cancer Institute, University of Utah, Salt Lake City, UT, United States

**Keywords:** inflammatory bowel disease, lycium barbarum polysaccharides, macrophage polarization, STAT1, STAT6

## Abstract

Disruption of colonic homeostasis caused by aberrant M1/M2 macrophage polarization contributes to the development of inflammatory bowel disease (IBD). Lycium barbarum polysaccharide (LBP) is the primary active constituent of traditional Chinese herbal *Lycium barbarum L.*, which has been widely demonstrated to have important functions in regulating immune activity and anti-inflammatory. Thus, LBP may protect against IBD. To test this hypothesis, the DSS-induced colitis model was established in mice, then the mice were treated with LBP. The results indicated that LBP attenuated the weight loss, colon shortening, disease activity index (DAI), and histopathological scores of colon tissues in colitis mice, suggesting that LBP could protect against IBD. Besides, LBP decreased the number of M1 macrophages and the protein level of Nitric oxide synthase 2(NOS2) as a marker of M1 macrophages and enhanced the number of M2 macrophages and the protein level of Arginase 1(Arg-1) as a marker of M2 macrophages in colon tissues from mice with colitis, suggesting that LBP may protect against IBD by regulating macrophage polarization. Next, the mechanistic studies in RAW264.7 cells showed that LBP inhibited M1-like phenotype by inhibiting the phosphorylation of STAT1, and promoted M2-like phenotype by promoting the phosphorylation of STAT6. Finally, immunofluorescence double-staining results of colon tissues showed that LBP regulated STAT1 and STAT6 pathways *in vivo*. The results in the study demonstrated that LBP could protect against IBD by regulating macrophage polarization through the STAT1 and STAT6 pathways.

## 1 Introduction

Over the past decade, as a chronic relapsing disease, the incidence and prevalence of IBD have increased steadily (Raman and Ghosh, 2019; Kaplan and Windsor, 2021). For IBD, there is currently no very effective treatment, and the development of new and effective drugs is urgently required. Clinically, IBD is often accompanied by symptoms of abdominal pain, diarrhea, the loss of weight, and hematochezia (Danese et al., 2014). Activated immune system and excessive inflammatory response are considered to be the most important hallmarks of IBD, and immune cells play a key regulatory function in this process. Macrophages in particular are thought to play a crucial role in the onset and outcome of IBD (Rauh et al., 2005; Krausgruber et al., 2011), and considered to be the most attractive target for immune-mediated IBD therapy.

Macrophages mainly include two polarization phenotypes, the M1-like phenotype and the M2-like phenotype. M1 macrophages are induced by LPS and IFN-γ with the signatures of the expression of the NOS2 gene, and the production of IL-6, TNF-α, IL-1β, and other cytokines (Bouguen et al., 2011). During the onset of IBD, M1-like macrophages in the intestinal tract secrete inflammatory factors to maintain their pro-inflammatory capacity (Tian et al., 2021). M2-like macrophages are mostly induced by IL-4 and IL-13 with the signatures of the expression of the Arg-1, Ym1, and Fizz1 genes and the production of cytokines including IL-10 (Mills, 2015). With the development of IBD, M2-like macrophages in the intestinal tract increase production of anti-inflammatory factors and inhibit secretion of pro-inflammatory factors that is critical for recovery from IBD (Na et al., 2019). LPS and IFN-γ (hereafter, “LPS/IFN-γ”) is considered the most classical inducer of M1 macrophages (Das et al., 2015). As for M2 polarization, we adopted the most widely used inducer IL-4 and IL-13 (hereafter, “IL-4/IL-13”).

Many previous researches have revealed that transcription factors play a key role in regulating macrophage polarization. Transcription factors associated with M1-like macrophage polarization mainly include STAT1 (Wu et al., 2021), NF-κB (Oeckinghaus et al., 2011), and IRF5 (Krausgruber et al., 2011). In contrast, STAT6, C/EBPβ, and IRF4 play a key role in M2-like macrophage polarization (Satoh et al., 2010). Transcription factors STAT1 and STAT6 closely regulate macrophage polarization. It has been reported that IFN-γ and LPS can activate the STAT1 pathway and promote M1-like polarization of macrophage (Hu and Ivashkiv, 2009). Whereas, IL-4/IL-13 activates the STAT6 pathway (Darnell et al., 1994; Kis et al., 2011) and promotes M2-like polarization of macrophage (Ding et al., 2019).

For thousands of years, *Lycium barbarum L.* has been a very important Chinese herbal medicine with the function of nourishing Yin and reinforce Qi. In modern medical research, clinical manifestations related to IBD are generally considered to be the result of deficiency of Qi and Yin (Zhao et al., 2014). In recent years, the polysaccharides derived from Angelica sinensis and Rheum tanguticum, had been found to be protective against diarrhea, the inflammation of colonic mucosal and ulcer in animal models (Liu et al., 2008; Wong et al., 2008). However, whether LBP, the polysaccharides extracted from *L. barbarum L.*, has a protective effect on IBD has not yet been revealed.

Studies have found that LBP has the function of regulating immune activity and anti-inflammatory (Tian et al., 2019; Fu et al., 2021). It exhibits immunomodulatory functions on many immune cells including macrophages and target dendritic cells (Zhang et al., 2015). It has been reported that the extract of *L. barbarum L.* can modulate the immune activity by promoting nitric oxide production and cytokine secretion in RAW264.7 cell (Lin et al., 2008). Therefore, whether LBP has a protective effect on IBD through its potential immunomodulatory function is worth further exploration.

In this study, the protective role of LBP in colitis mice induced by DSS and the underlying mechanism of this protective effect *in vitro* and *in vivo* were investigated. We found that LBP could alleviate IBD by regulating macrophage polarization by the STAT1 and STAT6 pathways.

## 2 Materials and methods

### 2.1 Cell culture, macrophage polarization, LBP treatment

RAW264.7 cells, a macrophage line of mouse, was obtained from Cell Bank of Academy of Sciences (Shanghai, Chain). After detection of mycoplasma-free contamination, the cells were seeded in DMEM medium (Gibco, Carlsbad, CA, United States) with 10% fetal bovine serum, 200 μg/ml L-glutamine, 100 U/mL penicillin, 100 μg/mL streptomycin and cultured at 37°C, 5% CO_2_.

M1 macrophages were generated by stimulation with 100 ng/mL LPS (*Escherichia coli*, Sigma-Aldrich, United States) and 10 ng/mL IFN-γ (Sino Biological, Shanghai, China) for 24 h. RAW264.7 cells were induced with 10 ng/mL IL-4 and 10 ng/mL IL-13 (both purchased from Sino Biological) for 24 h to generate of M2 macrophages. LBP and LPS/IFN-γ treated simultaneously RAW264.7 cells for 24 h, or LBP alone treated RAW264.7 cells for 24 h. Fludarabine and AS1517499 (both purchased from TargetMol, Shanghai, China) were employed to validate the STAT1and STAT6 signaling pathways.

### 2.2 Mice

The procedure of all animal experiment was approved by the Institutional Animal Care and Use Committee (IACUC) of Shaanxi Normal University (permit number: SCXK (Shan) 2021-002), China. The mice were kept in an environment with the controlled temperature, humidity, and light (23°C–24°C, 55%–65%, the cycle of 12/12 h light/dark) and provided with adequate water and food.

### 2.3 DSS-induced experimental colitis murine model

C57BL/6 male mice (22–27 g, 8 weeks old) were weighed. Then they were divided to five groups randomly (*n* = 5 in each group). There are group A, NC (negative control); group B, NC + LBP 200 mg/kg; group C, DSS; group D, DSS + LBP 100 mg/kg (low-dose), and group E, DSS + LBP 200 mg/kg (high-dose), respectively. The mice from groups A and B were given drinking water for 0–9 days. The mice from groups C, D, and E were administered 2.5% DSS (MW50000; MP Biomedicals, Irvine, CA, United States) in water of drinking for 0–7 days to build the experimental colitis murine model.

LBP was purchased from Solarbio Science and Technology Co., Ltd. (Cat No. SP9311, Beijing, China), derived fromLycium fruits with water extract-alcohol precipitation method. Total sugar content was determined by the phenol-sulfuric acid method using glucose as standard (Dubois et al., 1956). The content of total sugar was 93.2%. The 200 and 100 mg/kg LBP were used in animal experiments according to the previous literatures (Zhang et al., 2020; Xie et al., 2021). The mice in groups D, B, and E were given every day 100 mg/kg or 200 mg/kg LBP (dissolved in 1×PBS) by gavage at a fixed time from the second day of the DSS perform, and the mice in groups A and C were given the same amount of 1×PBS by gavage until the ninth day.

Each mouse of every group was under close, daily observation for the body weight, the consistency of stool and hematochezia. The DAI was calculated based on the loss of weight, the consistency of stool and hematochezia, based on the scoring standards in the literature (Shon et al., 2015). The mice were euthanized on day nine.

### 2.4 Cell viability assay

In RAW264.7 cells, the influence of LBP on the viability was tested by the CCK-8 kit (HB-CCK8-1, Hanbio Biotechnology, Shanghai, China). Briefly, 5 × 10^3^ cells/well in 100 μL of cell suspension were plated in the 96-well plate. 200, 400, 800, or 1,600 μg/mL LBP were used to treated the cells. After incubating for 24 h at 37°C, 5% CO_2_, discarded the medium, then 100 μL of fresh medium and 10 μL of CCK8 reagent were added for another 4 h incubation followed by the absorbance reading at 450 nm using the microplate reader (Thermo, United States). The cell viability of each group was calculated according to the following formula: the cells viability (%) = (OD_LBP_ -OD _blank_)⁄(OD _control_ -OD _blank_) × 100.

### 2.5 *q*PCR

The Total RNA from various groups of RAW264.7 cells was extracted with TRIzol ^®^ reagent (Takara, Dalian, China). Then 500 ng total RNA was used for the reverse transcription experiment with the Prime Script™ RT Reagent Kit (Takara). In a system of ABI 7900HT Fast Real-Time PCR, the PCR Kit (SYBR green), (QIAGEN, Hilden, Germany) was used to perform the *q*PCR experiment. Each test was repeated at least three times. The mRNA expression level of all genes tested was normalized with *ß*-actin as the standard using the 2^−△△Ct^ method. The main primers for *q*PCR are shown in Table 1.

**TABLE 1:** The sequences of the primers used for *q*PCR in the study.

Primer	Sequence (5′–3′)
Mouse NOS2 forward	CAA​CAT​CAG​GTC​GGC​CAT​CAC​T
Mouse NOS2 reverse	ACC​AGA​GGC​AGC​ACA​TCA​AAG​C
Mouse Ym1 forward	AGA​AGG​GAG​TTT​CAA​ACC​TGG​T
Mouse Ym1 reverse	GTC​TTG​CTC​ATG​TGT​GTA​AGT​GA
Mouse IL-6 forward	CCT​TCC​TAC​CCC​AAT​TTC​CAA​T
Mouse IL-6 reverse	GCC​ACT​CCT​TCT​GTG​ACT​CCA​G
Mouse IL-10 forward	GGT​TGC​CAA​GCC​TTA​TCG​GA
Mouse IL-10 reverse	GGG​GAG​AAA​TCG​ATG​ACA​GC
Mouse IFN-γ forward	AGC​AAC​AGC​AAG​GCG​AAA​A
Mouse IFN-γ reverse	CTGGACCTGTGG GTTGTTGA
Mouse β-actin forward	ACA​GAG​CCT​CGC​CTT​TGC​CGA​T
Mouse β-actin reverse	GAC​CCA​TGC​CCA​CCA​TCA​CGC

### 2.6 Immunofluorescence staining

The cells were washed and fixed using paraformaldehyde at a concentration of 4%, then incubated with the diluted primary antibodies at 4°C overnight. The primary antibodies used are anti-NOS2 and anti-Arg-1 (rabbit polyclonal, 1:300, both from Proteintech, Chicago, IL, United States), p-STAT1 (rabbit polyclonal, 1:200, Affinity, Shanghai, China). After washing of cells with 1×PBS, cells were incubated with goat anti-rabbit IgG Dylight 594 or Dylight 488 (1:500, both from Abbkine, Wuhan, China) for 1 h in the dark. Representative pictures were captured with a Zeiss Imager M2 microscope and were analyzed using Image J software (version: v1.8.0).

### 2.7 Hematoxylin and eosin (H&E) and immunofluorescence double-staining

Mice in the five groups were anesthetized with ether, and their colons were removed and fixed with paraformaldehyde at a concentration of 4%. Then, the tissues were paraffin-embedded to make wax blocks and cut into 5 mm sections. Next, the H&E staining were performed after the sections were dried. The specific staining procedure was performed according to our previous staining procedure (Mao et al., 2017).

Immunofluorescence double-staining in mouse colon tissue was performed. Paraffin-embedded slides were performed the quenching of dewaxed, rehydrated, antigen repair, the block of endogenous peroxidase activity and non-specific antigens. Next, the slides were co-incubated with the following primary antibodies: anti-F4/80 (rabbit polyclonal, 1:10,000), anti-NOS2, and anti-Arg-1 (rabbit polyclonal, 1:1,500) (all purchased from Servicebio Co., Ltd., Wuhan, China); and p-STAT1, p-STAT6 (rabbit polyclonal, 1:200) (both from Affinity, Shanghai, China). Then the slides were co-incubated with the corresponding HRP labeled secondary antibody for 50 min at room temperature. Next, the corresponding opal tyramines signal amplification (TSA) stain working solution (Cat: G1226, purchased from Servicebio Co., Ltd., Wuhan, China) was dripped onto the tissue to ensure complete tissue coverage, and incubated for 10 min at room temperature. After the second run, DAPI was used to stain nucleus. Representative pictures of H&E and immunofluorescence double-staining were captured with a Zeiss Imager M2 microscope. The number of positive cells was counted in five randomly selected areas under fluorescence microscope.

### 2.8 Western blot

Mouse colon tissues or RAW264.7 cells were added with an appropriate amount of RIPA buffer, followed by protease inhibitors and phosphatase inhibitors, and lysed on ice for 40 min. Then, nuclear and cytoplasmic proteins from RAW264.7 cells were separated using the Nuclear and Cytoplasmic Extraction Kit. After protein quantification, the same amount of protein was added to each gel well, and separated by 10% resolving gel and 5% stacking gel and transferred onto PVDF membranes. After blocking with 5% BSA for 2–3 h, incubated PVDF membranes with NOS2, Arg-1 (rabbit polyclonal, 1:500), Lamin A/C (rabbit polyclonal, 1:1,000) (all purchased from Proteintech, Chicago, IL, United States); and p-STAT1, STAT1, p-STAT6, and STAT6 (rabbit polyclonal, 1:500) (all from Affinity, Shanghai, China); *ß*-actin (rabbit polyclonal, 1:1,000) (from Santa Cruz, Dallas, TX, United States) at 4°C overnight. Next, the membranes were disposed with HRP-labeled Goat IgG antibody (anti-rabbit, 1:10,000) (from Zhong Shan, Beijing, China) for 1 h. Then the membranes were washed with 1×PBST, and incubated in a photoluminescence solution for imaging. The bands of the protein were analyzed by Image J software (version: v1.8.0).

### 2.9 Statistical analysis

Comparisons between the two groups were performed using Student’s t-test. One-way ANOVA was used to compare three or more groups. Statistical analysis of the obtained data was performed using GraphPad Prism 8.0 software, and the data are reported as the mean ± SD. In all figures, *p* < 0.05 indicates that the difference was statistically significant.

## 3 Results

### 3.1 LBP significantly reduced the severity of DSS-induced colitis in mice

**FIGURE 1:**
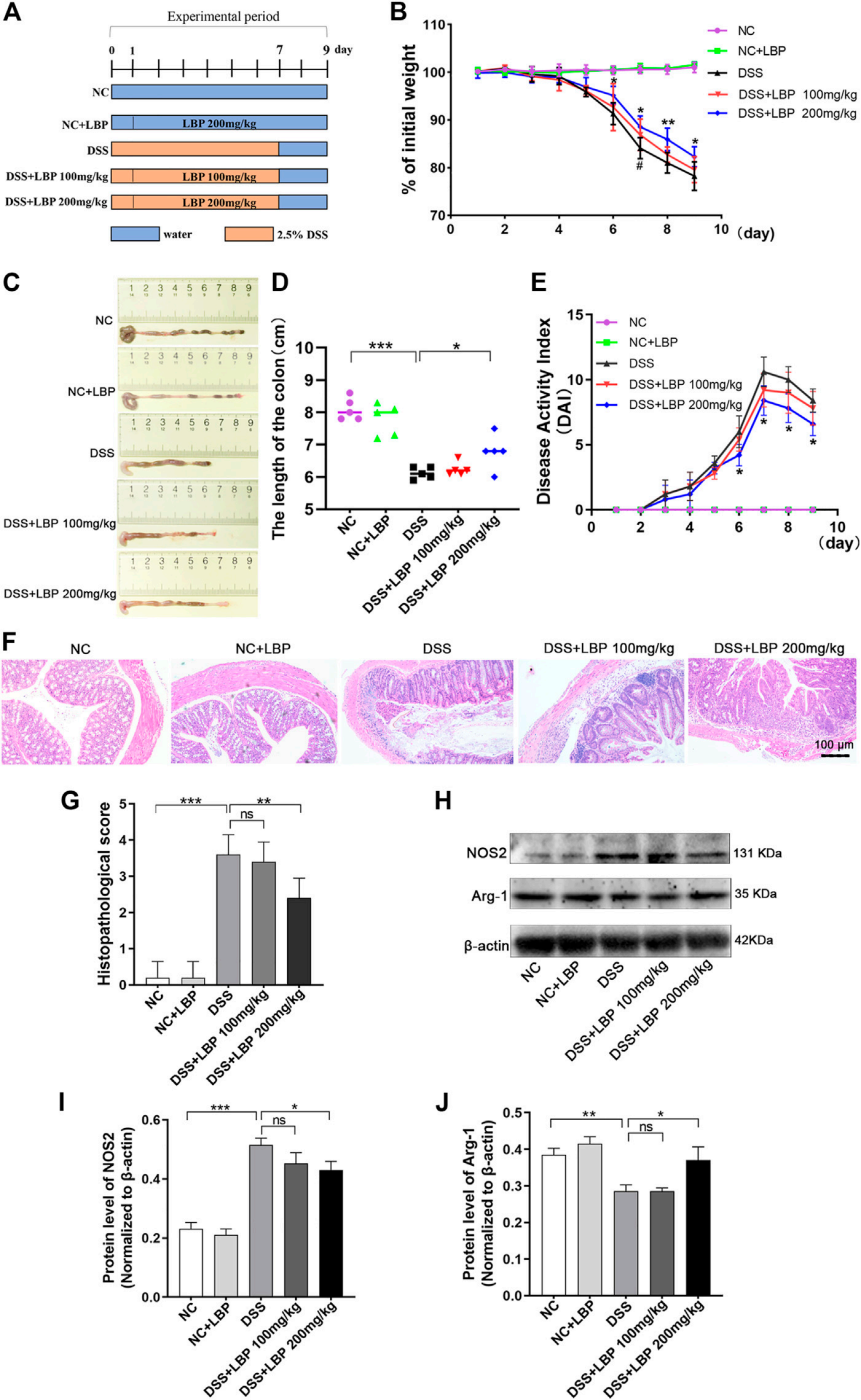
LBP obviously attenuates the severity of colitis in DSS-induced C57BL/6 mice. **(A)** Experimental procedure for building colitis model using 2.5% DSS. **(B)** The weight of mice in the five groups was assessed daily. **(C)** Representative pictures of colon from mice in five groups; the length of the colon was measured on day nine. **(D)** The quantification analysis of colon length of mice from five group. **(E)** The evaluation of DAI score of mice from five groups. **(F)** Representative histopathological images of colon from mice in each group. **(G)** The statistical analysis of H&E pathological score of colons from each group. **(H)** Western blot results of NOS2 and Arg-1 in tissues of the colon from five groups of mice. **(I)** The quantification of NOS2 in Figure 1H. **(J)** The quantification of Arg-1 in Figure 1H. *p < 0.05, **p < 0.01, ***p < 0.001, the DSS + LBP 200 mg/kg group vs. the DSS group; ^#^ *p* < 0.05, the DSS + LBP 100 mg/kg group vs. the DSS group.

To study whether LBP has a protective role for IBD, a murine colitis model of DSS-induced was established. The execution process of the animal experiment is shown in Figure 1A. In the process of the experiment, the DSS-treated mice exhibited obvious symptoms of the weight loss, diarrhea, and bloody stool. In contrast with the DSS group, the high dose of LBP (DSS + LBP 200 mg/kg) group was found to significantly protect from weight loss (Figure 1B) and atrophy of the colon (Figures 1C, D), and reduce the DAI score in mice with colitis (Figure 1E). However, the treatment group with low dose of LBP (DSS + LBP 100 mg/kg) was also found to obviously protect from the weight loss in mice relative to the DSS group on day seven after DSS induction (Figure 1B).

On the basis of assessing the symptoms of colitis in mice, the histological morphology of the colon tissue was further investigated. The results indicated that the DSS + LBP 200 mg/kg group showed significantly less disordered tissue structure, and the infiltration of inflammatory cells and histological damage as significantly lower, as was the histological score in contrast with the DSS group (Figures 1F, G).

In addition, a great number of macrophages were found in the colon tissue of mice DSS- induced. The previous researches have shown that the polarization of macrophages played a dominant role in the onset and outcome of IBD (Zhu et al., 2016; Zhou et al., 2019). To further investigate whether LBP alleviated IBD by regulating macrophage polarization, the protein level of NOS2 and Arg-1 were examined by Western blot in colon tissues. It was found that the protein level of NOS2 in the DSS + LBP 200 mg/kg group was markedly decreased in contrast with the DSS group (Figures 1H, I), while the protein level of Arg-1 was obviously increased in contrast with the DSS group (Figures 1H, J), suggesting that LBP may protect against IBD by regulating macrophage polarization.

**FIGURE 2:**
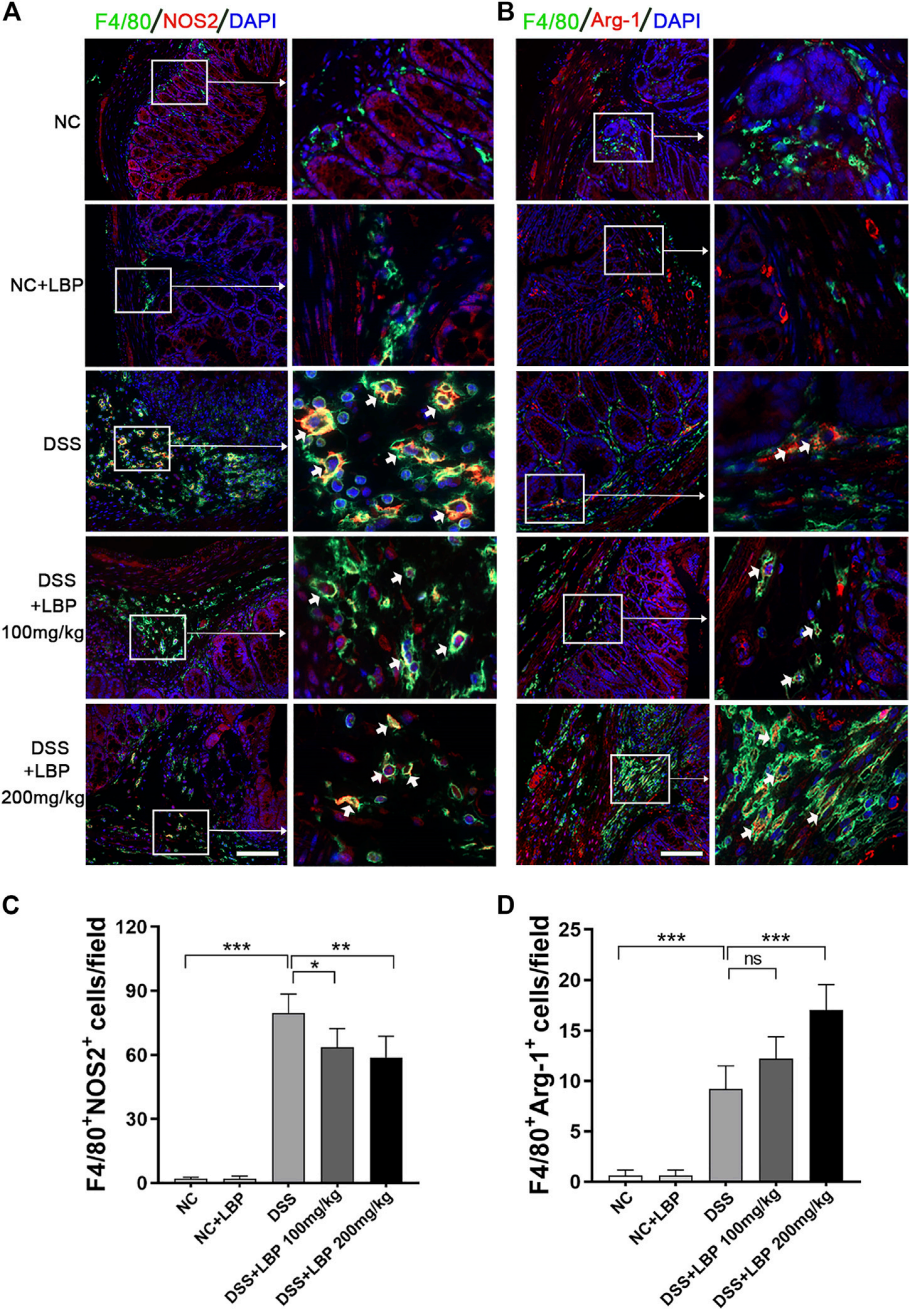
LBP treatment modulates the plasticity of intestinal macrophages. **(A)** Representative immunofluorescence images of F4/80 (green color) and NOS2 (red color) in colon tissues from five groups of mice, and cell nucleus were stained with DAPI (blue). The right panel is the inset image from the corresponding region from the left panel. **(B)** Representative immunofluorescence images of F4/80 (green color) and Arg-1 (red color) in colon tissues from five groups of mice, and cell nucleus were stained with DAPI (blue). The right panel is the inset image from the corresponding region from the left panel. **(C)** The number of F4/80^+^ NOS2^+^ cells in Figure 2A were quantified. **(D)** The number of F4/80^+^ Arg-1^+^ cells in Figure 2B were quantified. Scale bar, 100 µm. Significant differences are indicated as *p < 0.05, **p < 0.01; ***p < 0.001. Ns, no significance.

Next, immunofluorescence double-staining was performed to further detect whether LBP treatment affects the changes in the quantity of M1 and M2 macrophages in the colon tissues of mice with colitis. The results showed that 100 and 200 mg/kg LBP treatment both significantly reduced the quantity of F4/80^+^ NOS2^+^ M1 macrophages, and the effect of 200 mg/kg LBP was more obvious (Figure 2A). 200 mg/kg LBP treatment obviously increased F4/80^+^ Arg-1^+^ M2 macrophages (Figure 2B) in the colonic tissue of mice with colitis. The quantitative results of the number of F4/80^+^ NOS2^+^ M1 and F4/80^+^ Arg-1^+^ M2 macrophages in Figures 2A, B were shown in Figures 2C, D. The results further proved that LBP could protect against IBD by regulating macrophage polarization.

### 3.2 LBP regulated macrophage polarization by the STAT1 and STAT6 pathways *in vitro*

The results above suggested that LBP could alleviate the symptoms of DSS-induced colitis by potentially regulating the polarization of macrophages. In order to further research the potential mechanisms of LBP regulating macrophage polarization, the experiments were further performed using RAW264.7 macrophage cell line for the purpose *in vitro*.

**FIGURE 3:**
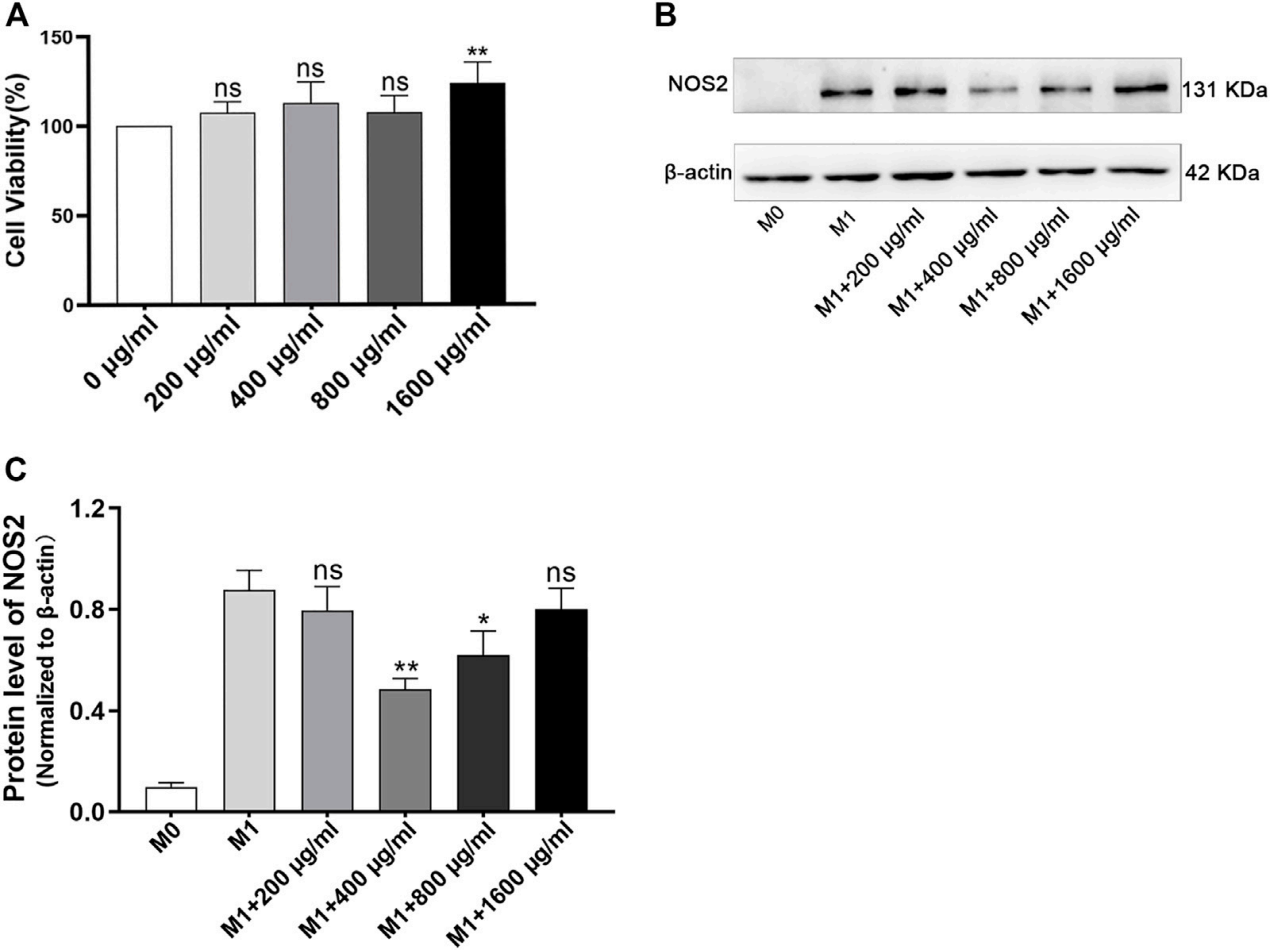
The effect of LBP on cell viability in RAW264.7 cells and the selection of optimal concentration of LBP for macrophage polarization. **(A)** The RAW264.7 cells were cultured with different dosages of LBP for 24 h, and the cell viability was tested. **p < 0.01 vs. 0 μg/mL group (100% cell viability). Ns, no significance. **(B)** The protein level of NOS2 was detected in RAW264.7 treated with LBP and LPS/IFN-γ for 24 h via Western blot. **(C)** The quantification of NOS2 in Figure 3B in each group. *p < 0.05, **p < 0.01 vs. the M1 group. Ns, no significance.

To optimize the dose of LPB *in vitro* experiment, the different doses of LBP were tested *in vitro* by the evaluation of the viability of RAW264.7 cells by CCK8 assay. The results indicated that all concentrations of LBP tested were found to have no toxicity to the RAW264.7 cells (Figure 3A). Then, the influence of different concentrations of LBP on the protein level of NOS2 in RAW264.7 cells with M1 phenotype was detected. It was found that 400 μg/mL LBP had a significant role in reducing the expression of NOS2 (Figures 3B, C). Therefore, 400 μg/mL of LBP was selected as the final working concentration for subsequent experiments.

**FIGURE 4:**
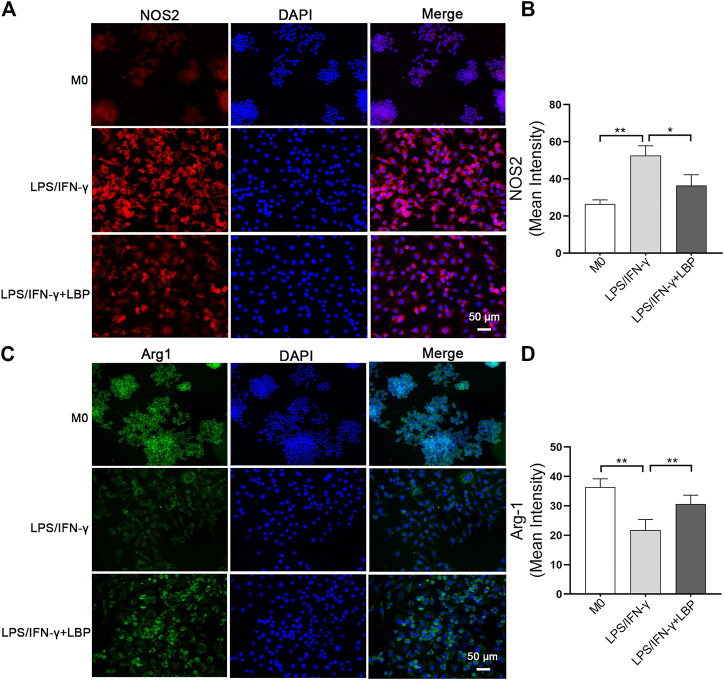
The influence of LBP on the expression of NOS2 and Arg-1 in RAW264.7 cells was investigated by immunostaining. **(A)** Immunofluorescence staining of NOS2 (red) in different groups. **(B)** The quantification of the mean fluorescence intensity of NOS2 in Figure 4A. **(C)** Immunofluorescence staining of Arg-1 (green) in different groups. **(D)** The quantification of the mean fluorescence intensity of Arg-1 in Figure 4C. Significant differences are indicated as *p < 0.05, **p < 0.01.

Then, 400 μg/ml as the treatment concentration, the phenotypic experiments on the effect of LBP on the polarization of macrophage were performed in RAW264.7 cells. The immunofluorescence results revealed that the stimulation of LPS/IFN-γ obviously increased the expression of NOS2, however, this elevated expression was significantly suppressed in the presence of LBP (Figure 4A). The quantification of the mean fluorescence intensity of Figure 4A each group is shown in Figure 4B. Differently, the expression of Arg-1 was obviously decreased after the stimulation of LPS/IFN-γ, however, the expression of Arg-1 was increased when RAW264.7 cells were subjected to both LBP and LPS/IFN-γ (Figure 4C). The quantification of the mean fluorescence intensity of Figure 4C each group is shown in Figure 4D.

**FIGURE 5:**
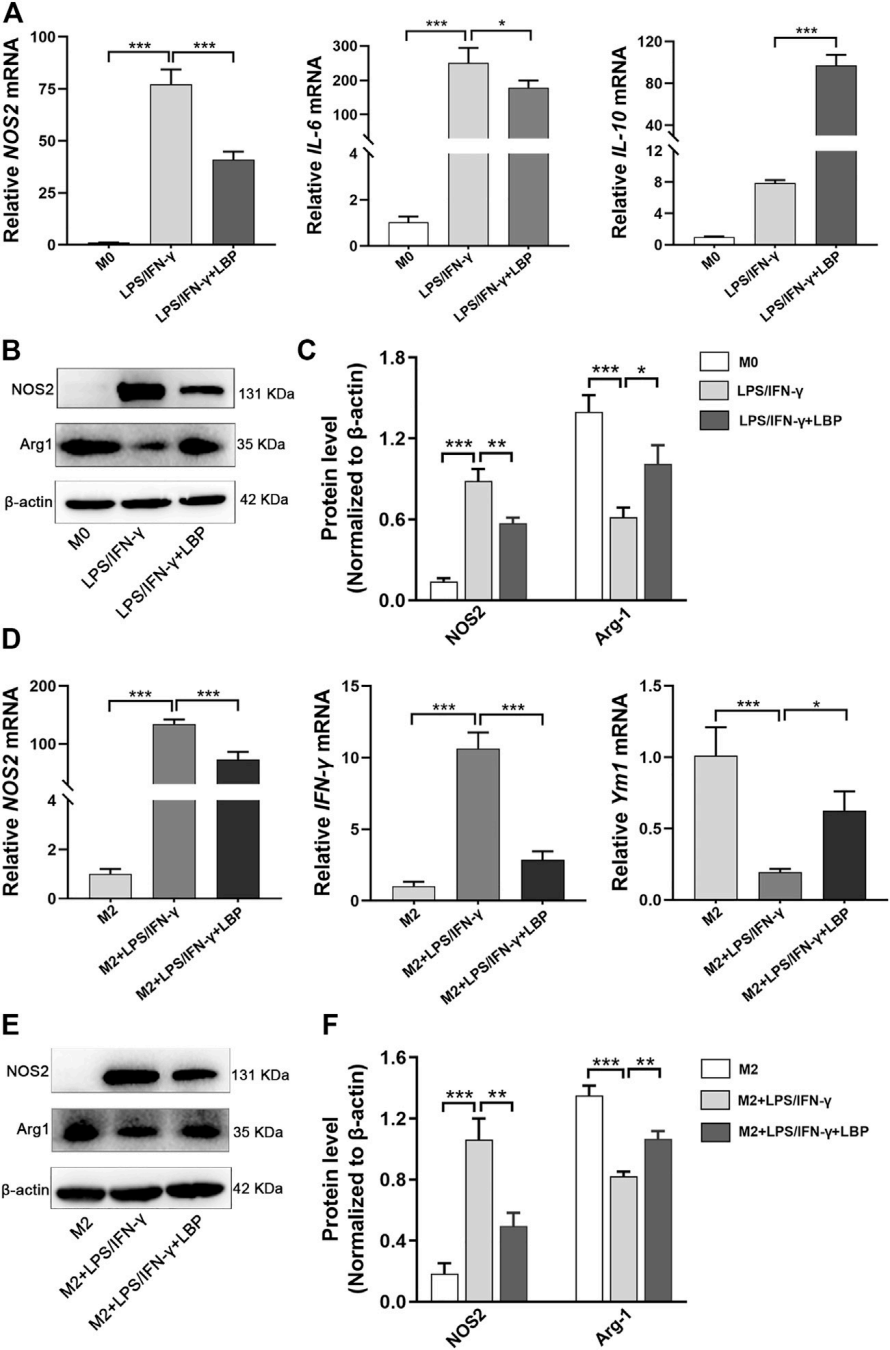
The influence of LBP on the expression of the related genes involving the polarization of macrophage was tested by *q*PCR and Western blot. **(A)** The *q*PCR results of NOS2, IL-6, and IL-10 in the M0, LPS/IFN-γ, and LPS/IFN-γ + LBP groups. **(B)** Western blot results of NOS2, Arg-1, and *ß*-actin in the M0, LPS/IFN-γ, and LPS/IFN-γ + LBP groups. **(C)** The quantification of the protein bands of NOS2 and Arg-1 in Figure 5B. **(D)** The *q*PCR results of NOS2, IFN-γ, and Ym1 in the M2, M2 + LPS/IFN-γ, and M2 + LPS/IFN-γ + LBP groups. **(E)** Western blot results of NOS2, Arg-1, and *ß*-actin in the M2, M2 + LPS/IFN-γ and M2 + LPS/IFN-γ + LBP groups. **(F)** The quantification of the protein bands of NOS2 and Arg-1 in Figure 5E. Significant differences are indicated as *p < 0.05, **p < 0.01, ***p < 0.001.

To further explore the effect of LBP on macrophage polarization, the mRNA and protein levels of macrophage polarization-related genes were examined in RAW264.7 cells. The results revealed that the expression of NOS2 and IL-6 were obviously increased after stimulation using LPS/IFN-γ, while the expressions of both were significantly decreased after treatment with LBP (Figure 5A). The expression of IL-10 was increased after stimulation with LPS/IFN-γ + LBP compared with no LBP treatment (Figure 5A). The result of Western blot demonstrated that the protein level of NOS2 was obviously advanced, and the protein level of Arg-1 was markedly reduced after stimulation with LPS/IFN-γ. Treatment with LBP resulted in higher Arg-1 expression and lower NOS2 expression (Figure 5B). The quantification of protein bands in each group is shown in Figure 5C.

Next, IL-4/IL-13 were used to induce M2 polarization and stimulated M2 RAW264.7 cells using LPS/IFN-γ to observe the transition from M2 to M1 with or without LBP. The result of a *q*PCR showed that after LPS/IFN-γ treatment of M2 RAW264.7 cells, the mRNA level of NOS2 and IFN-γ were obviously enhanced, while the expression of Ym1 was markedly attenuated. However, in the presence of LBP, the mRNA level of NOS2 and IFN-γ were obviously reduced, and expression of Ym1 was increased (Figure 5D). Western blot results showed that M2 RAW264.7 cells after stimulation with LPS/IFN-γ showed higher NOS2 expression and lower Arg-1 expression. In the presence of LBP, the elevated expression of NOS2 was suppressed, whereas the expression of Arg-1 was significantly elevated (Figure 5E). The quantification of protein bands in each group is shown in Figure 5F. Thus, LBP can inhibit the repolarization of RAW264.7 cells from M2 to M1, which is beneficial for maintaining the M2-like phenotype.

**FIGURE 6:**
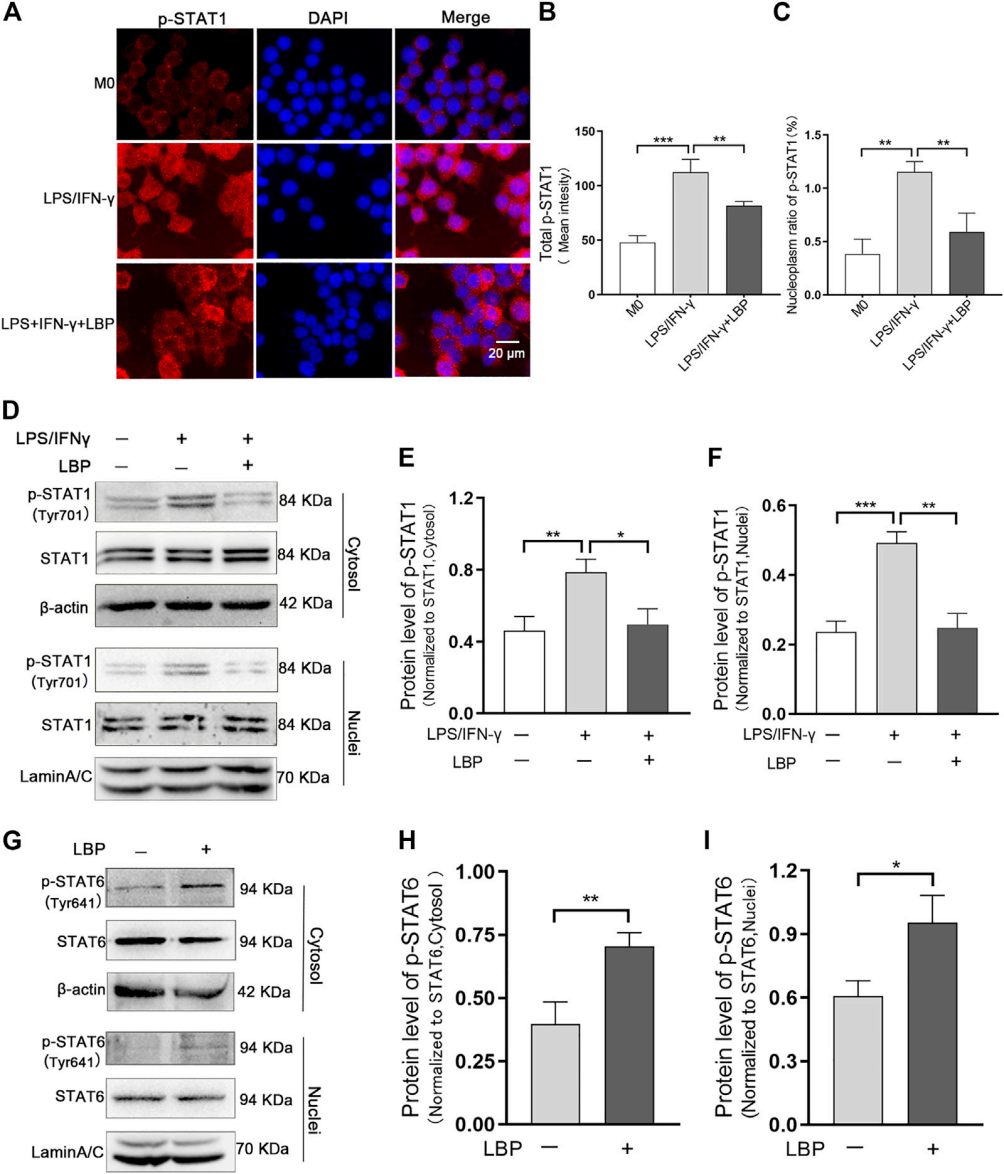
LBP regulates macrophage polarization through the STAT1 and STAT6 pathways. **(A)** Immunofluorescence staining of p-STAT1 (red) in RAW264.7 cells in M0, LPS/IFN-γ, and LPS/IFN-γ +LBP groups. **(B)** The quantification result of mean fluorescence intensity of total p-STAT1 in Figure 6A. **(C)** The quantification result of mean fluorescence density nucleoplasm ratio of p-STAT1in Figure 6A. **(D)** Western blot results of p-STAT1 in the cytosol and nuclei of RAW264.7 cells after stimulation with LPS/IFN-γ, with or without LBP treatment. **(E)** The quantification of the Western blot bands of p-STAT1 in the cytosol in Figure 6D. **(F)** The quantification of the Western blot bands of p-STAT1 in the nuclei in Figure 6D. **(G)** Western blot results of p-STAT6 in the cytosol and nuclei of LBP-treated and untreated RAW264.7 cells. **(H)** The quantification of Western blot bands of p-STAT6 in the cytosol in Figure 6G. **(I)** The quantification of Western blot bands of p-STAT6 in the nuclei in Figure 6G. Significant differences are indicated as *p < 0.05, **p < 0.01, ***p < 0.001.

It has been documented that STAT1 and STAT6 signaling pathways involves the polarization of M1/M2-like macrophage. To investigate whether LBP affects macrophage polarization by STAT1 and STAT6 signaling pathways, the effect of LBP on the STAT1 and STAT6 pathways was investigated in RAW264.7 cells. First, we detected the effect of LBP on p-STAT1 by cellular immunofluorescence (Figure 6A). The results of mean fluorescence intensity of total p-STAT1 (Figure 6B) and mean fluorescence intensity nucleoplasm ratio (Figure 6C) revealed that the protein level of total p-STAT1 and p-STAT1 in the nuclei were obviously increased after LPS/IFN-γ treatment, suggesting the activation of STAT1 signaling. In the presence of LBP, the protein level of total p-STAT1 and p-STAT1 in the nuclei were obviously decreased.

Western blot results indicated that the protein level of p-STAT1 in the cytosol and nuclei of RAW264.7 cells were obviously increased after LPS/IFN-γ treatment. When RAW264.7 cells were subjected to both LBP and LPS/IFN-γ, the expression of p-STAT1 in the cytosol (Figures 6D, E) and nuclei (Figures 6D, F) was markedly reduced.

LBP was found to be beneficial in maintaining the M2-like phenotype (Figure 5). Furthermore, the activation of the STAT6 signal pathway is required for maintaining the M2-like phenotype (Zhou et al., 2020). To test whether LBP maintained M2-like phenotype through the STAT6 signaling pathway, the protein level of p-STAT6 in the cytosol and nuclei of RAW264.7 cells with or without LBP treatment was tested via Western blot. The results proved LBP did increase the expression of p-STAT6 in the cytosol and nuclei (Figure 6G). The quantification of the protein bands of p-STAT6 in the cytosol and nuclei was shown in Figures 6H, I. At the same time, the level of p-STAT1 was detected in RAW264.7 cells with or without LBP treatment, and the results showed that treating RAW264.7 cells with LBP alone did not affect the level of p-STAT1 in the cytosol (Supplementary Figure S1A, B) and nuclei (Supplementary Figure S1A, C).

**FIGURE 7:**
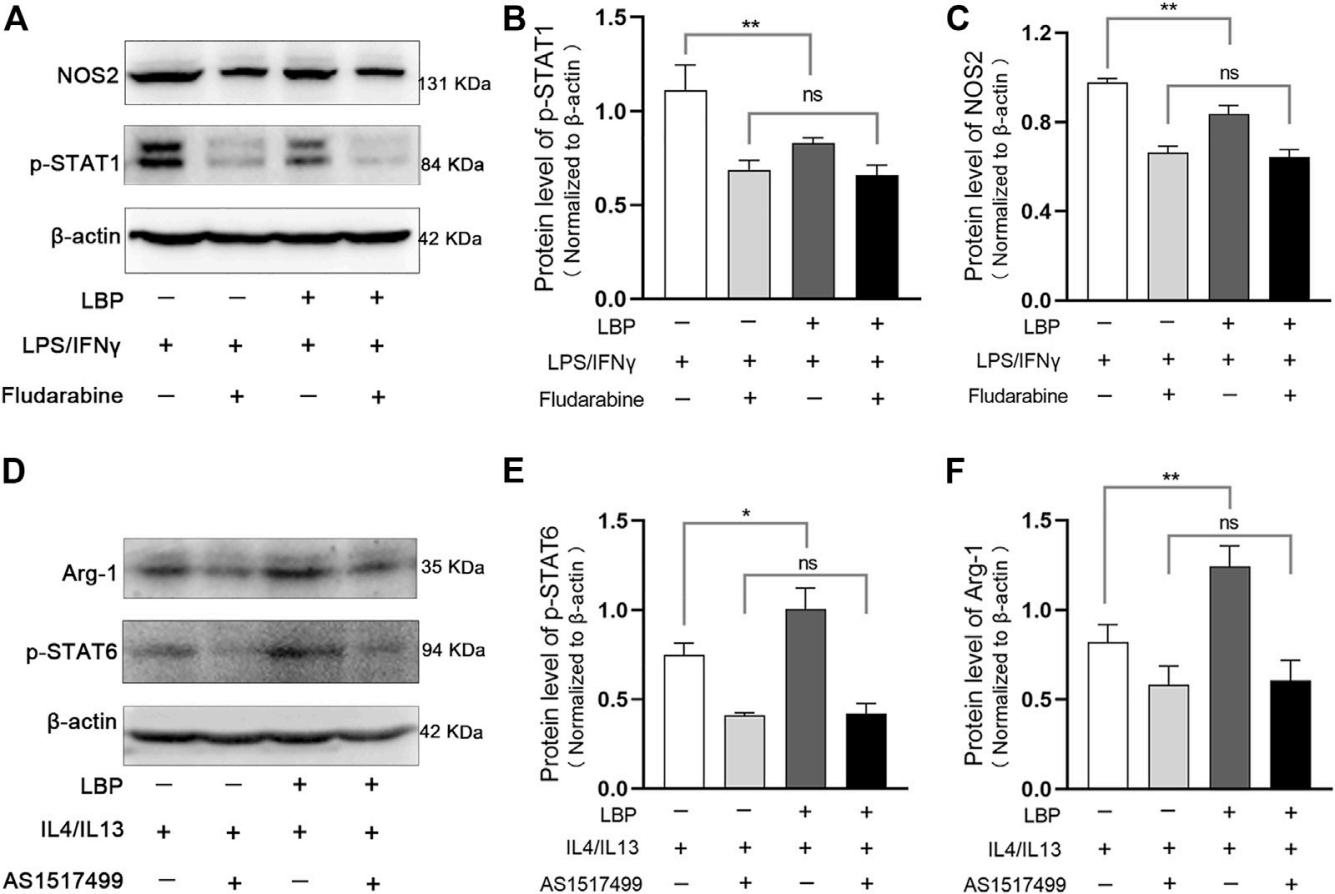
LBP regulates M1/M2 macrophage polarization through the STAT1 and STAT6 pathways. **(A)** RAW264.7 cells were stimulated with LPS/IFN-γ, then treated with LBP or Fludarabine alone or together. The treated cells were collected for Western blot. Representative Western blot images of p-STAT1, NOS2, and *ß*-actin. **(B)** The quantification of the Western blot bands of p-STAT1 in Figure 7A. **(C)** The quantification of the Western blot bands of NOS2 in Figure 7A. **(D)** RAW264.7 cells were stimulated with IL4/IL13, then treated with LBP or AS1517499 alone or together. The treated cells were collected for Western blot. Representative Western blot images of p-STAT6, Arg-1, and *ß*-actin. **(E)** The quantification of the Western blot bands of p-STAT6 in Figure 7D. **(F)** The quantification of the Western blot bands of Arg-1 in Figure 7D. Significant differences are indicated as *p < 0.05, **p < 0.01. Ns, no significance.

Subsequently, we investigated whether the downregulation effect of LBP on LPS/IFN-γ-induced M1 macrophages depends on the STAT1 pathway. For this purpose, a potent inhibitor of p-STAT1 (Fludarabine) was administrated in LPS/IFN-γ-stimulated RAW264.7 cells with or without LBP, and the protein level of p-STAT1 and NOS2 were detected by Western blot. The results showed that LBP could significantly decreased the level of p-STAT1 and NOS2, suggesting that LBP inhibits the M1 phenotype by inhibiting the STAT1 signaling pathway. Moreover, when Fludarabine dramatically reduced the protein level of p-STAT1, the inhibitory effect of LBP on M1 macrophage phenotype was significantly disrupted (Figure 7A). The quantification of the protein bands of p-STAT1 and NOS2 was shown in Figures 7B, C.

We also investigated whether the effect of LBP on M2 macrophages depends on the STAT6 signaling pathway by using an effective inhibitor of p-STAT6 (AS1517499) in IL4/IL13-stimulated RAW264.7 cells with or without LBP. Western blot analysis showed that LBP could significantly increase the protein level of p-STAT6 and Arg-1, suggesting that LBP promotes the M2 phenotype by promoting the STAT6 signaling pathway. Moreover, the effect of LBP on promoting M2 phenotype disappeared when p-STAT6 was significantly inhibited (Figure 7D). The quantification of the protein bands of p-STAT6 and Arg-1 was shown in Figures 7E, F. Taken together, these data indicate that LBP regulates M1/M2 macrophage polarization through STAT1 and STAT6 signaling pathways.

### 3.3 LBP regulated the STAT1 and STAT6 pathways *in vivo*

**FIGURE 8:**
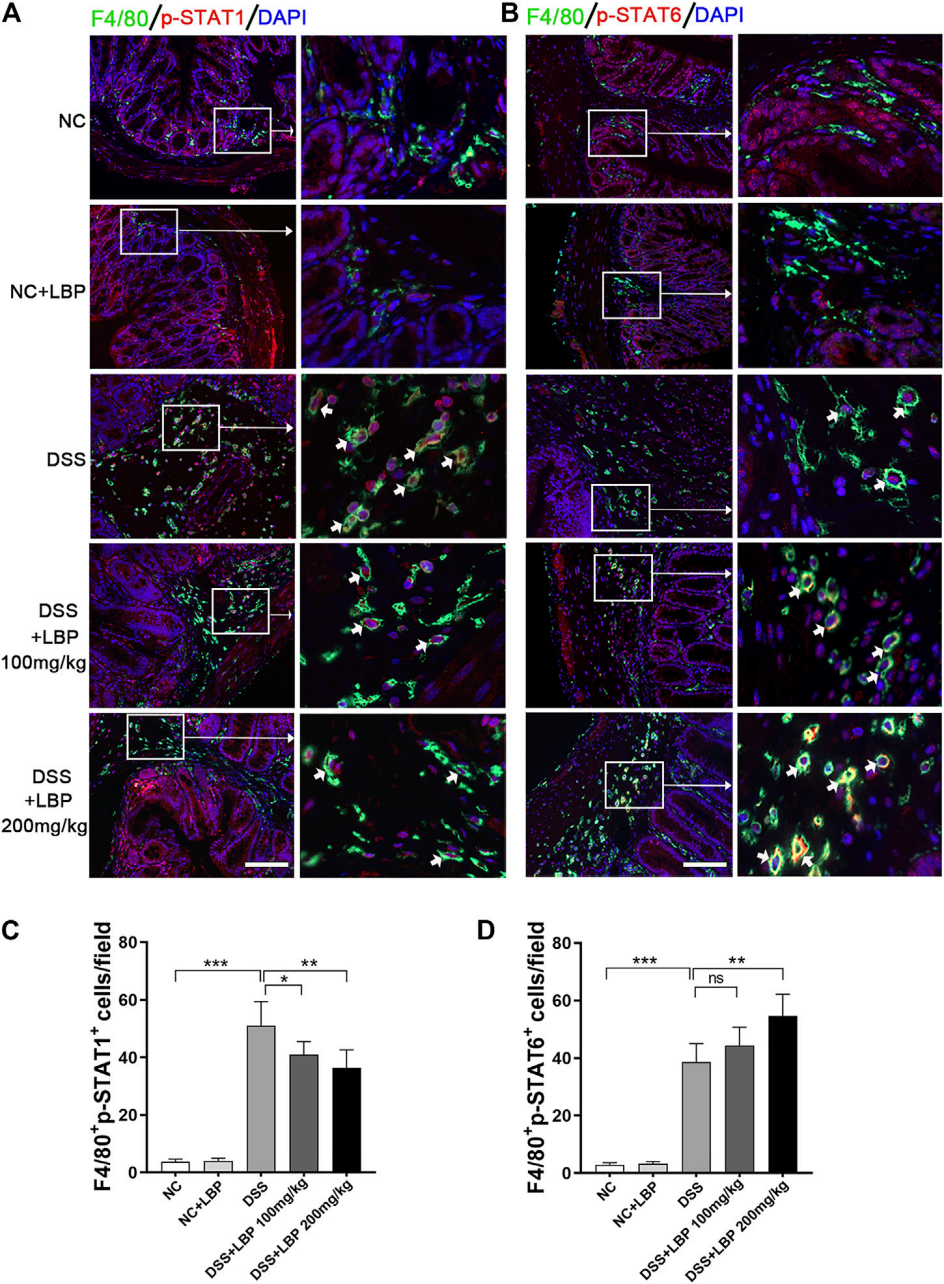
LBP regulates the STAT1 and STAT6 pathways in the colon tissue of mice DSS- induced. **(A)** Representative immunofluorescence images of F4/80 (green color) and p-STAT1 (red color) in colon tissues from five groups of mice, and cell nucleus were stained with DAPI (blue). The right panel is the inset image from the corresponding region from the left panel. **(B)** Representative immunofluorescence images of F4/80 (green color) and p-STAT6 (red color) in colon tissues from five groups of mice, and cell nucleus were stained with DAPI (blue). The right panel is the inset image from the corresponding region from the left panel. **(C)** The number of F4/80^+^ p-STAT1^+^ cells in Figure 8A were quantified. **(D)** The number of F4/80^+^ p-STAT6^+^ cells in Figure 8B were quantified. Scale bar, 100 µm. Significant differences are indicated as *p < 0.05, **p < 0.01; ***p < 0.001. Ns, no significance.

We had demonstrated *in vitro* that LBP regulated M1/M2 macrophage polarization by modulating the STAT1 and STAT6 pathways, and also found that LBP could regulate macrophage polarization in DSS-induced colitis mice by the detection of the expression of M1 or M2 marker in colon tissue (Figures 1H–J; Figure 2). In order to further verify whether LBP regulated M1/M2 macrophage polarization by STAT1 and STAT6 signaling pathways *in vivo*, the immunofluorescent double-staining of F4/80 and p-STAT1 or p-STAT6 were performed to detect the level of p-STAT1 and p-STAT6 in intestinal macrophages of mice. The results indicated that the quantity of F4/80^+^ p-STAT1^+^ cells in the DSS + LBP 100 mg/kg group and the DSS + LBP 200 mg/kg group was obviously reduced in contrast with the DSS group (Figure 8A), while the quantity of F4/80^+^ p-STAT6^+^ cells in DSS + LBP 200 mg/kg group was obviously increased in contrast with the DSS group (Figure 8B). The quantification of results of the number of F4/80^+^ p-STAT1^+^ cells, and F4/80^+^ p-STAT6^+^ cells in Figures 8A, B were revealed in Figures 8C, D. This result showed that LBP regulated STAT1 and STAT6 pathways *in vivo*, which was consistent with the results *in vitro*.

**FIGURE 9:**
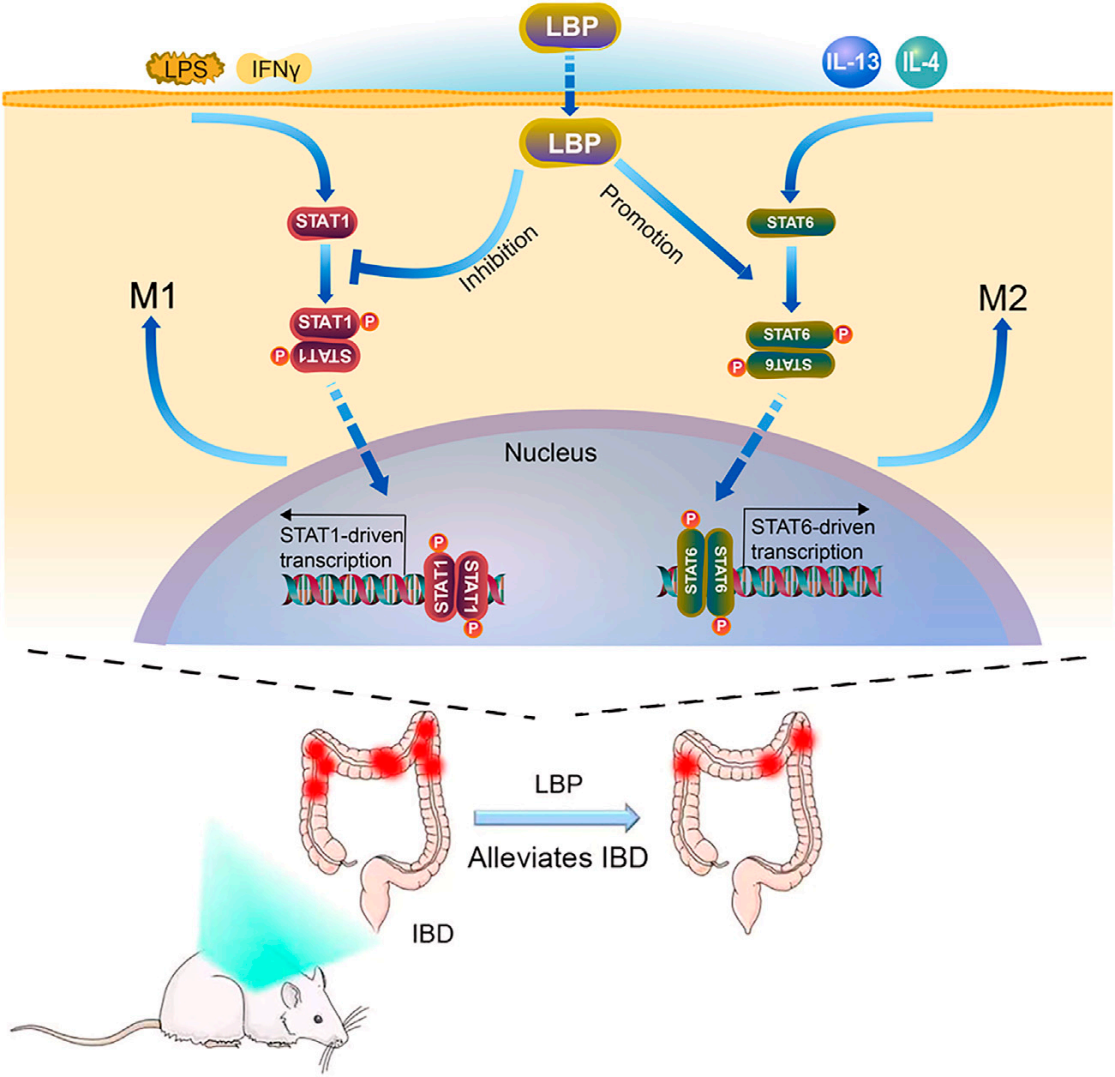
The Model diagram of LBP in alleviating IBD by regulating M1/M2 macrophage polarization by STAT1 and STAT6 pathways. *In vitro*, LBP inhibits the polarization of M1 macrophages by suppressing the phosphorylation of STAT1, and promotes the polarization of M2 macrophages by promoting the phosphorylation of STAT6. *In vivo*, LBP regulates the polarization of macrophages to protect against DSS-induced IBD by regulating the STAT1 and STAT6 pathways.

Based on the results *in vitro* and *in vivo*, the mechanisms of LBP regulating macrophage polarization was summarized in Figure 9. In the STAT1 and STAT6 pathways, LPS and IFN-γ can activate the STAT1 pathway, which promotes M1-like polarization of macrophage; IL-4 and IL-13 activate the STAT6 signaling pathway, which promotes M2-like polarization of macrophage. LBP inhibited M1 macrophage polarization by reducing the level of p-STAT1 and promoted M2 macrophage polarization by increasing the level of p-STAT6 *in vivo* and *in vitro*, and resulting in the alleviation of DSS-induced IBD in mice.

## 4 Discussion

As a chronic recurrent disease, the characteristic of IBD is frequent inflammation response in the intestinal tissue. Its pathogenesis has not been fully elucidated, and both genetic and environmental factors may contribute to epithelial barrier dysfunction (Xu et al., 2014). IBD is crucial characterized by the abnormal inflammatory response (Rosenzwajg et al., 2019). It is generally believed that an imbalance in M1/M2 macrophages is strongly correlated with the outcome of IBD (Zhou et al., 2019; Du et al., 2021). At present, effective cure for IBD is still absent. Comprehensive treatment methods such as drugs, biological agents, and surgery are commonly used in clinical practice (Shen et al., 2019), despite of the poor effectiveness. Therefore, it is essential to develop new IBD drugs that have higher effectiveness and with no safety concerns.

Traditional herbal medicines have been used for the treatment of colitis in China for hundreds of years. *Lycium barbarum L.* is a traditional Chinese herbal, and its main bioactive component is LBP, which has anti-inflammatory, antioxidant, and immune regulation activity (Qi et al., 2022; Sun et al., 2022). Some previous studies have found that LBP attenuates LPS-induced inflammation by triggering the pyruvate kinase M2(PKM2) degradation and altering the differentiation of macrophages (Huan et al., 2021). LBP also attenuates collagen II-induced arthritis in mice by altering inflammatory mediators in serum and bone (Liu et al., 2015). To observe the effects of LBP on IBD, we established a colitis model induced by DSS in mice, mimicking the human IBD symptoms. These results in this study showed that LBP significantly reduced the loss of weight, improved colon shortening and DAI, and decreased the pathological scores of colon tissue in DSS-induced colitis mice, indicating that LBP could alleviate DSS-induced colitis symptoms.

How does LBP suppress inflammation and reduce DSS-induced colitis symptoms? Many studies have demonstrated that there are a great many of macrophages in the intestinal tissue, and the establishment of IBD is closely related to the phenotypic switch of these macrophages (You et al., 2016; Rabbi et al., 2017). Given the close relationship between macrophage polarization and IBD, we examined indicators of macrophages polarization in the colon of colitis mice and found that LBP treatment promoted macrophages to the M2-like phenotypes and inhibited macrophages to the M1-like phenotypes in colon tissues. Thus, we believe that LBP may protect IBD by regulating the polarization of macrophages. To test this idea, RAW264.7 cells were selected to explore the influence of LBP on macrophage polarization. The results of different concentrations of LBP on the protein level of NOS2 in RAW264.7 cells with M1 phenotype indicated that 400 μg/mL LBP had a significant role in reducing the expression of NOS2. Surprisingly, we found that 1,600 μg/mL LBP had no significant effect on the protein level of NOS2. Why high concentration of LBP has no effect on the protein level of NOS2 needs to be further explored. The results revealed that the appropriate concentration of LBP could inhibit M1 polarization of macrophage *in vitro.*

Das et al. (2015) reported that macrophages can reverse their polarized phenotypes depending on the chemokine milieu, enabling the complete repolarization of macrophages from M2 to M1. To further explore the effect of LBP on the polarization, we introduced the treatment of LBP in the process of M2 repolarization, and found that M2 macrophages could be repolarized to the M1-like phenotype after the stimulation of LPS/IFN-γ *in vitro*. LBP could inhibit the repolarization of M2 to M1, which is beneficial to maintaining the M2-like phenotype.

Many signaling molecules such as STAT1, STAT6, IRF4, IRF5, C/EBPβ regulate M1/M2 polarization, determining the fate of macrophages. STAT1 and STAT6 pathways play a critical role in this progression of macrophage polarization (Yin et al., 2018; Ding et al., 2019) and have been demonstrated to be closely related to the progression of IBD (Tian et al., 2021). In murine IBD model, STAT6-dependent macrophage can activate Wnt signaling to promote mucosal repair of mice (Cosin-Roger et al., 2016). Coriolus versicolor suppresses IBD by inhibiting the expression of STAT1 associated with IFN-γ expression (Lim, 2011). According to the effect of LBP on the macrophage phenotype and results from previous studies on STAT1 and STAT6 pathways involvement in IBD progression (Lim, 2011; Salas et al., 2020; Tian et al., 2021), we speculate that LBP might affect macrophage polarization through the STAT1 and STAT6 pathways to protect against IBD.

In our work, we confirmed that LBP could inhibit the phosphorylation of STAT1 and suppress the M1 macrophage polarization, and promote the phosphorylation of STAT6 and the M2 macrophage polarization (Figure 9). By using specific inhibitors of p-STAT1and p-STAT6, we further demonstrated that the regulatory effect of LBP on macrophage polarization is mediated through the STAT1 and STAT6 signaling pathways. We validated this result *in vivo*, and the results showed that LBP-treated DSS-induced mouse colon tissue expressed higher p-STAT6 and lower p-STAT1, indicating that LBP could regulate the STAT1 and STAT6 pathways *in vivo* (Figure 8).

## 5 Conclusion

In summary, LBP protects against IBD by regulating the polarization of macrophage through the STAT1 and STAT6 pathways. The study offers new perspectives on the mechanism of LBP regulating inflammation and a theoretical basis for developing LBP as a new treatment for IBD.

## Data Availability

The original contributions presented in the study are included in the article/Supplementary Material, further inquiries can be directed to the corresponding author.
