# Supplementary material for: Lycium barbarum polysaccharide alleviates dextran sodium sulfate-induced inflammatory bowel disease by regulating M1/M2 macrophage polarization via the STAT1 and STAT6 pathways
Source: Front Pharmacol. 2023 Apr 18;14:1044576. doi: 10.3389/fphar.2023.1044576 (PMC10151498; doi:10.3389/fphar.2023.1044576)
Supplement: Supplementary file 1 [file Image1.pdf]

Figure S1.

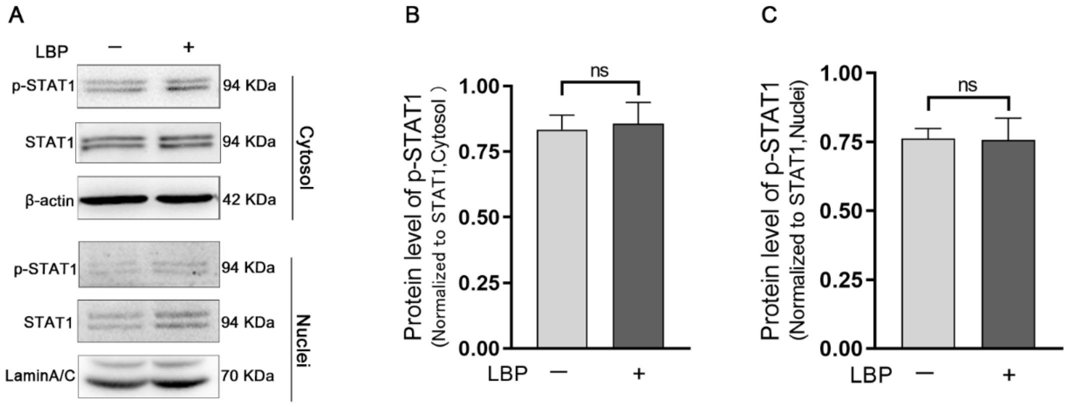

Figure S1. The influence of LBP on the expression of p-STAT1 in the cytosol and nuclei of RAW264.7 cells with or without LBP treatment.

(A) Western blot results of p-STAT1 in the cytosol and nuclei of RAW264.7 cells with or without LBP treatment. The quantification of the protein bands of p-STAT1 in the cytosol **(B)** and nuclei **(C)** of RAW264.7 cells with or without LBP treatment.
